# Human gut derived-organoids provide model to study gluten response and effects of microbiota-derived molecules in celiac disease

**DOI:** 10.1038/s41598-019-43426-w

**Published:** 2019-05-07

**Authors:** Rachel Freire, Laura Ingano, Gloria Serena, Murat Cetinbas, Anthony Anselmo, Anna Sapone, Ruslan I. Sadreyev, Alessio Fasano, Stefania Senger

**Affiliations:** 1 Mucosal Immunology and Biology Research Center and Center for Celiac Research and Treatment, Department of Pediatrics, Massachusetts General Hospital, Boston, MA USA; 2 Harvard Medical School, Boston, MA USA; 3 Department of Molecular Biology, Cancer Center and Center for Regenerative Medicine, Massachusetts General Hospital, Boston, MA USA; 4 Present Address: PatientsLikeMe, Inc., Cambridge, MA USA; 5 Present Address: Translational Research and Early Clinical (TREC), GI, Takeda Pharmaceuticals International Co., Boston, MA USA; 6 European Biomedical Research Institute of Salerno (EBRIS), Salerno, Italy

**Keywords:** Intestinal stem cells, Coeliac disease

## Abstract

Celiac disease (CD) is an immune-mediated disorder triggered by gluten exposure. The contribution of the adaptive immune response to CD pathogenesis has been extensively studied, but the absence of valid experimental models has hampered our understanding of the early steps leading to loss of gluten tolerance. Using intestinal organoids developed from duodenal biopsies from both non-celiac (NC) and celiac (CD) patients, we explored the contribution of gut epithelium to CD pathogenesis and the role of microbiota-derived molecules in modulating the epithelium’s response to gluten. When compared to NC, RNA sequencing of CD organoids revealed significantly altered expression of genes associated with gut barrier, innate immune response, and stem cell functions. Monolayers derived from CD organoids exposed to gliadin showed increased intestinal permeability and enhanced secretion of pro-inflammatory cytokines compared to NC controls. Microbiota-derived bioproducts butyrate, lactate, and polysaccharide A improved barrier function and reduced gliadin-induced cytokine secretion. We concluded that: (1) patient-derived organoids faithfully express established and newly identified molecular signatures characteristic of CD. (2) microbiota-derived bioproducts can be used to modulate the epithelial response to gluten. Finally, we validated the use of patient-derived organoids monolayers as a novel tool for the study of CD.

## Introduction

Celiac disease (CD) is an immune-mediated disorder characterized by an autoimmune enteropathy triggered by gluten intake in genetically predisposed subjects carrying HLA-DQ2 or HLA-DQ8 haplotypes^1^. About 1% of the general population is affected by the disease^2^. The enteropathy is characterized by villous atrophy, crypt hyperplasia, and swelling of the lamina propria with infiltration of immune cells, including neutrophils and lymphocytes^3^. Gluten-specific and tissue transglutaminase (tTG) antibodies are expressed during the acute phase^2^.

Extensive studies have been conducted on gluten, the known trigger for CD. Gluten is composed of gliadins and other storage proteins found in wheat, barley, and rye^1^. Gliadin’s family has been identified as responsible for CD pathogenesis in multiple ways, exerting cytotoxic, immunomodulatory, and gut-permeating activities on the intestinal mucosa^4^.

Based on current data, it is hypothesized that CD onset is preceded by the following sequence of events: after oral intake, undigested gliadin peptides trigger the release of zonulin, leading to increased intestinal permeability^5,6^. The disrupted barrier allows translocation of gliadin’s peptides to the lamina propria and subsequent interaction with macrophages and other immune cells^4,7^. Gliadin peptides promote IL8 and IL15 secretion from enterocytes, leading to recruitment of neutrophils^8^ and intraepithelial lymphocytes^9^. Finally, the interaction of T cells with gliadin’s peptide-presenting cells in a pro-inflammatory milieu leads to the abrogation of oral tolerance and activation of the Th1/Th17 adaptive immune response^10^.

The chain of events as hypothesized implies that the interaction between the host and the trigger is necessary and sufficient, suggesting that the onset of CD occurs at the time of the first encounter, namely the introduction of solid, gluten-containing food to at-risk infants. However, epidemiological data suggest that CD onset can occur at any age, even decades after the introduction of gluten into the diet^11,12^. Therefore, other factor(s) must be at play to dictate “if and when” an individual genetically at risk for CD loses tolerance to gluten. Novel evidence suggests that the gastrointestinal tract microbiota may play a role in the pathogenesis of CD^13,14^. A proof-of-concept study by Sellitto and co-authors^15^ established that infants who were genetically predisposed to CD and went on to develop the disease expressed a dysbiotic microbiota during the preclinical phase of CD. This was characterized by a decreased representation of *Bacteriodetes*, a high abundance of *Firmicutes*, and a peak and the subsequent drop of *Lactobacillus* species. These changes were concurrent with alterations of the microbiota metabolome signature regarding the relative abundance of butyrate and lactate levels^15^. Of note, both butyrate and lactate have been shown to exert a relevant role in regulating the ratio between FoxP3 spliced isoforms in T cells and consequent activation of the Th17-driven immune response in CD^16^.

Although major efforts have been undertaken to understand the adaptive immune component associated with the physiopathology of CD, little is still known about the early steps leading to loss of gluten tolerance. The lack of a reliable *in vivo* animal model for CD has hampered our scientific progress, which has been mainly generated by *in vitro* studies on whole biopsies, or on immortalized or cancer cell lines^17^. Questions remain about antigen trafficking, activation of the innate immune response, and the development of crypt hyperplasia in a person with a genetic background at risk for CD. Furthermore, to date, no studies have defined whether and how gut microbiota composition and derived bioproducts could mechanistically contribute to CD onset. Thanks to recent development of new techniques, it is now possible to generate organoids from human intestine, an important tool for a patient-derived *in vitro* model^18,19^.

Therefore, in this study, we aimed at harnessing this technology to generate and validate the use of intestinal organoids from patients to investigate the contribution of the intestinal epithelium in CD pathogenesis. We compared the global gene expression in organoids derived from CD and non-celiac (NC) patients to identify differences relevant to the enteropathy. We established a reliable tool to study intestinal epithelial cell permeability, immune function, and epithelium regeneration. Moreover, based on our hypothesis that peculiar profiles of microbiota and their derived bioproducts are mechanistically linked to modifications of gut mucosal functions, we evaluated the effect of bacterial bioproducts in modulating the epithelium’s response to gliadin.

## Results

### RNA sequencing analysis in organoids reveals differences in gene expression potentially relevant to celiac disease pathogenesis

**Figure 1:**
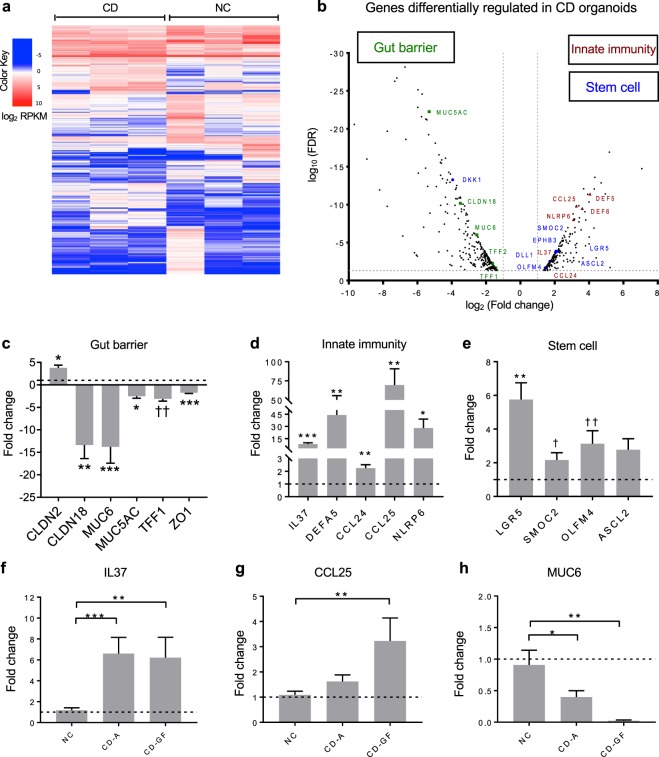
Differential gene expression profiles in active celiac epithelial organoids and human whole duodenal biopsies. (**a**) Heatmap representing RNA-seq expression values (log_2_ RPKM) for the genes differentially expressed in organoids from patients with active celiac disease (CD, n = 3) compared to non-celiac controls (NC, n = 3). A color code from blue to red indicates low and high expression levels, respectively. (**b**) Volcano plot showing fold change (X axis in log_2_ scale) and statistical significance (FDR, Y axis in log_10_ scale) for differentially expressed genes (RNA-seq) in active celiac (CD, n = 3) compared to non-celiac (n = 3) organoids. Selected differentially expressed genes are highlighted and colored by functional categories associated with gut barrier function (green), innate immunity (red), and stem cell function (blue). (**c**–**e**) Validation by qRT-PCR of selected genes found differentially expressed in active celiac epithelial organoids by the RNA-seq analysis, belonging to relevant functional categories: gut barrier (**c**), innate immunity (**d**), and stem cell (**e**). Data represent average expression relative to NC control ± SEM; *p < 0.05, **p < 0.01, ***p < 0.001, two-side unpaired t-test; ^†^p < 0.05, ^††^p < 0.01, Mann-Whitney test; 12 to 18 replicates for n = 5 non-celiac and n = 5 active celiac organoids. (**f**–**h**) Gene expression assessed by qRT-PCR in human duodenal biopsies of non-celiac (NC, n = 6–11), celiac patients with active disease (CD-A, n = 17), and celiac patients in remission following a gluten-free diet (CD-GF, n = 5–6) to validate the organoid model. Data represent average expression relative to NC ± SEM. *p < 0.05, **p < 0.01, ***p < 0.001, Mann-Whitney test.

We generated and characterized epithelial organoids derived from duodenal biopsies of NC and CD patients. We aimed at comparing patterns of gene expression in the organoids using RNA sequencing (RNA-seq). Multivariate analyses revealed that the samples grouped together based on their respective diagnosis. While the active CD-derived organoids shared similar transcriptional signatures, the NC sample set appeared more heterogeneous (Fig. 1a). Nonetheless, we found 472 genes differentially expressed (fold change greater than 2, FDR < 0.05) between the two groups. Of them, 291 genes were downregulated and 181 were upregulated in CD compared to NC (Fig. 1b; Supplementary Table S1).

Although CD is an adaptive immune-mediated enteropathy, evidence suggests that changes associated with intestinal functions, including gut permeability and innate immunity, could play a pivotal role in CD onset^5,9,20^. Furthermore, crypt hyperplasia, a hallmark of CD that occurs during the acute phase, has been associated with the dysregulation of intestinal stem and progenitor cells^21^. To further investigate functional categories among genes that are differentially expressed in CD organoids, we performed DAVID gene ontology analysis (GO)^22,23^, gene set enrichment analysis (GSEA)^24^ as well as in-depth manual inspection of differentially expressed genes to identify candidates that could contribute to CD pathogenesis. Among differentially expressed genes, the greatest percentage clustered by terms “extracellular space” and extracellular region” in the category GO cellular component (GOTERM_CC) and “signal” and “secreted” in UP Keywords (Supplementary Table S2). Those genes included stem cell, glycocalyx genes and chemokines. Specifically, components of the WNT signaling pathway, including LGR5, OLFM4, SMOC2, ASCL2, EPHB3, and DLL1, were found upregulated in CD organoids, whereas DKK1, a WNT negative regulator^25^ was significantly downregulated (Fig. 1b; Supplementary Fig. S1). These data are consistent with an overrepresentation of actively proliferating cells, including stem and progenitor cells in CD organoids, and are in line with previous findings in celiac biopsies^21^.

We observed that genes associated with gut barrier functionality such as the pore-forming claudin 2 (CLDN2)^26^ and the essential scaffolding protein tight junction protein 1 (TJP1; also known as ZO1)^27^, were respectively up- and downregulated in CD organoids, albeit slightly below the two-fold threshold (fold change 1.9 and 1.55). These results are consistent with previous observations in CD biopsies^28,29^. Additionally, we identified novel genes that are significantly downregulated in CD organoids, including sealing tight junction protein CLDN18^26^ and some components of the mucus layer MUC6, MUC5AC, and trefoil factors (TFF1 and TFF2). In addition, genes associated with an innate immune response, such as the chemokines CCL24 and CCL25, the initiator of the inflammasome complex formation NLRP6, the alpha-defensins DEFA5 and DEFA6, and the anti-inflammatory cytokine IL37 were upregulated in organoids from CD patients (Fig. 1b).

To validate the RNA-seq dataset related to the three key biological functions outlined above, quantitative reverse transcription-PCR (qRT-PCR) was performed on a subset of the identified genes in a larger organoid sample set. We confirmed the upregulation of CLDN2 and downregulation of CLDN18, MUC6, MUC5AC, TFF1 and ZO1 in active CD organoids (Fig. 1c). Relatively to innate immunity associated genes, we confirmed the upregulation of all the evaluated genes: IL37, DEFA5, NLRP6, CCL24 and CCL25 (Fig. 1d). Finally, we evaluated the expression of the genes involved in stem cell function and found, consistent with RNA-seq data set, significant upregulation of LGR5, SMOC2 and OLFM4. ASCL2 was found upregulated, but not significantly (Fig. 1e).

Studies from our group and others have established that human organoids retain a gene expression program that recapitulates the expression of the tissue of origin, including a diseased state^19,30^. To substantiate these findings in CD organoids, we evaluated the expression of selected newly identified genes including IL37, CCL25, MUC6, CLDN18 and CCL24 in duodenum biopsies from NC, CD patients with active disease, and CD patients in remission following a gluten-free diet (GFD). Consistent with the expression in CD organoids, we found that IL37 and CCL25 were upregulated, whereas MUC6 was significantly downregulated in the CD biopsies of both active and in-remission patients (Fig. 1f–h). CCL24 was not differentially expressed, whereas CLDN18 was found upregulated in CD-A duodenum biopsies (Supplementary Fig. S1b).

### CD organoids proliferative defects suggest impaired repair functions of the stem cell compartment

**Figure 2:**
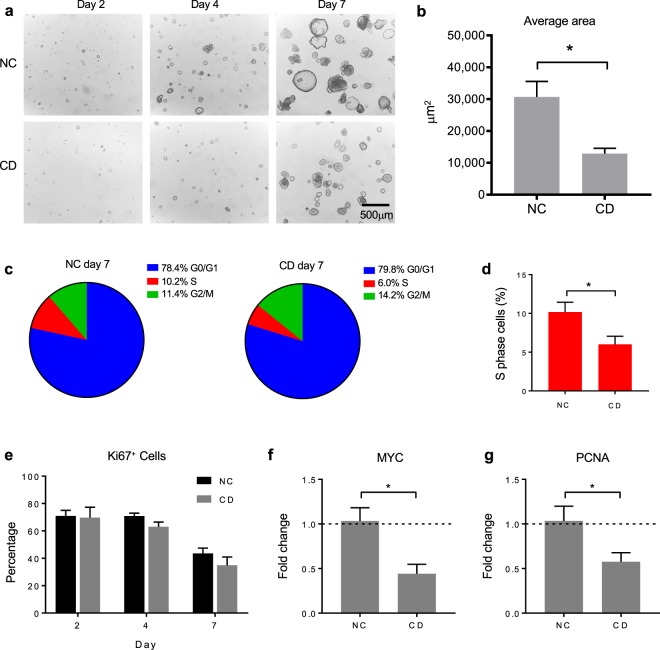
Development and proliferation of non-celiac and active celiac organoids over time. (**a**) Representative images of one non-celiac (NC) and one active celiac (CD) epithelial organoids’ growth in matrigel 2, 4 and 7 days after plating. Cells were seeded at 5*10^4^ cells/mL density. (**b**) Averaged area (μm^2^) of organoids measured from microscopic image of n = 4 non-celiac (NC) and n = 3 active celiac (CD) patients, 7 days after seeding. ImageJ was used to calculate the area of 42 to 132 organoids for NC and 69 to 129 organoids for CD. Total area of the field is equal to 1.17*10^7^ μm^2^. Data represent average ± SEM. *p < 0.05, Mann-Whitney test. (**c**) Pie charts representing the percentage of cells in G0/G1 phase (blue), S phase (red) and G2/M phase (green) in non-celiac (NC, n = 4) and active celiac (CD, n = 4) organoids as determined by propidium iodide staining after 7 days in culture. Cells percentages were calculated out of gated cells that excluded debris, doublets and apoptotic cells. (**d**) Percentage of cells in S phase in non-celiac (NC, n = 4) and active celiac (CD, n = 4) organoids as determined by propidium iodide staining after 7 days in culture. Data represent average ± SEM. *p < 0.05, Mann-Whitney test. (**e**) Percentage of proliferating cells established by Ki67^+^ marker in non-celiac (NC, n = 4) and active celiac (CD, n = 4) organoids over time (day 2, 4 and 7). Double positive Ki67^+^H3P^+^ cells were excluded because represented mitotic cells. (**f**,**g**) Gene expression assessed by qRT-PCR in non-celiac (NC, n = 4) and active celiac (CD, n = 3) organoids. Data represent average expression relative to NC ± SEM. *p < 0.05, Mann-Whitney test.

Histological features including crypt hyperplasia and blunting of the villi are the hallmark of active CD characterized by expansion of the immature-cell population and concurring reduction of the mature cells of the villus^31^. These pathological manifestations of the CD intestinal mucosa have been hypothesized to be the consequence of a faulty stem cell compartment that cannot compensate the tissue turnover and/or a consequence of increased epithelium apoptosis^32,33^. To establish the proliferative capabilities of the epithelium we seeded NC and active CD single cells in matrigel and recorded the growth of organoids over time (Fig. 2a). We found that after 7 days in culture, the average area of CD organoids was significantly smaller compared to NC controls (Fig. 2b). To investigate the cause of reduced organoids growth, we evaluated the cell distribution across different cell cycle phases over time by propidium iodide (PI) staining, followed by FACS analysis in NC and active CD. We found no significant difference after two days in culture (Supplementary Fig. S2a). By day 4 we observed a reduction in cells in S phase in CD (Supplementary Fig. S2b). By day 7 we found significantly less cells in S phase in CD compared to NC (Fig. 2c,d). To further corroborate the observed reduction of cells in S phase, we evaluated the percentage of actively proliferating Ki67^+^ cells by FACS analysis at days 2, 4 and 7. There was no difference by day 2 between the two samples sets, suggesting that a comparable number of proliferative cells were initially plated. Less Ki67^+^ cells in CD samples compared to respective controls was observed as a trend by day 4 and 7 (Fig. 2e). Consistently we found reduced expression of proliferation markers MYC and PCNA in CD samples by day 7 (Fig. 2f,g). To establish a contribution of apoptosis to the reduced growth of the CD organoids, we evaluated the sub-G0/G1 cell population obtained by PI staining^34^ as a measure of DNA fragmentation associated to apoptosis^35^, that did not identify with cell debris (Supplementary Fig. 2c,d). We observed comparable frequency of sub-G0/G1 among the two samples sets at all the analyzed time points (Supplementary Fig. S2e). We also evaluated the expression of pro-apoptotic markers TNF receptor TNFRSF25^36^ and pro-apoptotic gene TP53^37^, and we did not find any statistically significant difference, even if TNFRSF25 appeared modestly upregulated in CD (Supplementary Fig. S2f). Finally, we performed immunofluorescence of activated caspase-3 on CD and NC organoids at day 7 and we did not observe significant difference between the samples sets (Supplementary Fig. S2g).

### Development and functional characterization of gut organoid-derived monolayers from CD patients

Next, we aimed at developing organoid-derived monolayers to investigate potential physiological and functional differences between the two experimental groups. We first established the haptoglobin (HP) genotype of the organoids employed in this study. Haptoglobin encodes for two alleles, of which HP2 carries a duplication of the 3^rd^ and 4^th^ exons^38^. The allele HP2, encodes for the HP2 pre-haptoglobin also known as zonulin, that has been shown to be a physiological regulator of tight junctions and it is released upon gluten stimulation^39,40^. We found that all the organoids had at least one copy of the HP2 allele (HP2-1 or HP2-2 genotype). Two NC and three CD organoids carried two copies of the allele (HP2-2 genotype).

**Figure 3:**
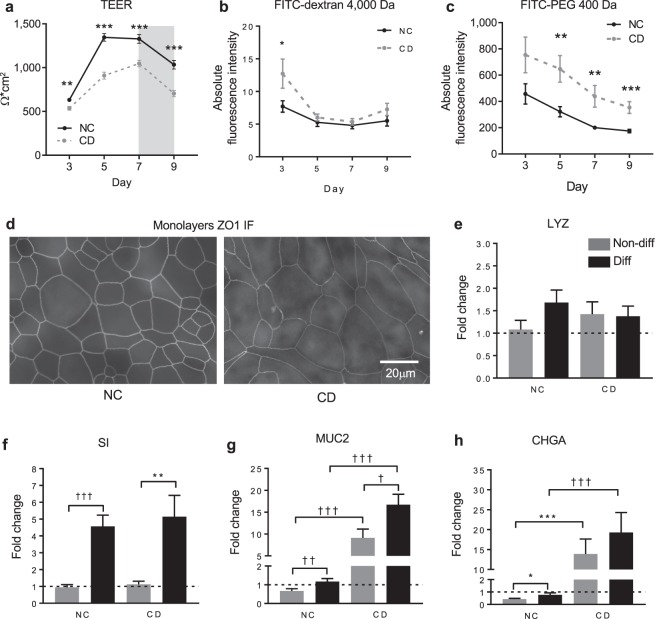
Development of non-celiac and celiac organoid-derived monolayers. (**a**) Transepithelial electrical resistance (TEER) measurement to evaluate development of the monolayers over time. Data represent average ± SEM. **p < 0.01, ***p < 0.001, two-side unpaired test; 149 to 234 replicates for n = 5 non-celiac (NC) and 156 to 253 replicates for n = 5 active celiac (CD) organoid-derived monolayers. (**b**,**c**) Absolute fluorescence intensity of FITC-dextran of 4,000 Da (**a**) and FITC-PEG of 400 Da (**b**) recovered in the basolateral side after 4 h of incubation. Data represent average ± SEM. (**b**) *p < 0.05, two-side unpaired t-test; 12 replicates for n = 4 non-celiac (NC) and n = 4 active celiac (CD) at each time point. (**c**) **p < 0.01, ***p < 0.001, two-side unpaired t-test; 12 and 9 replicates for n = 4 non-celiac (NC) and n = 3 active celiac (CD) at each time point. (**d**) Immunofluorescence staining of TJP1 (also known as ZO1) performed on one representative non-celiac (NC) and one active celiac (CD) monolayer at day 7 showing reduced deposition of the tight junction protein along the apical cell perimeter. (**e**–**h**) Gene expression assessed by qRT-PCR in organoid-derived monolayers cultured in two differentiation conditions (see Methods). LYZ (**e**), SI (**f**), MUC2 (**g**) and CHGA (**h**) evaluated the relative abundance of Paneth’s cells, enterocytes, goblet’s cells and neuroendocrine cells, respectively. Data represent average expression relative to NC non-differentiated ± SEM. *p < 0.05, **p < 0.01, ***p < 0.001, two-side unpaired t-test. ^††^p < 0.01, ^†††^p < 0.001 Mann-Whitney test; 20 and 16 replicates for n = 4 non-celiac (NC) and n = 3 active celiac (CD) patients.

We derived monolayers from NC and active CD-derived organoids, as previously described^19,41^. NC and CD monolayers developed with different kinetics based on transepithelial electrical resistance (TEER), with CD monolayers showing a significantly lower TEER at 3, 5, 7 and 9 days post seeding compared to NC (Fig. 3a). Furthermore, to evaluate the paracellular permeability in the developing monolayers, we employed two different molecular size neutral probes such as FITC-Dextran 4,000 Da and FITC-PEG 400 Da to measure their passage across the cell monolayers. While CD monolayers were more permeable to FITC-Dextran 4,000 Da at day 3 compared to NC monolayers, they showed comparable paracellular permeability by day 5 to day 9 (Fig. 3b). However, CD monolayers showed significantly higher permeability to the small FITC-PEG 400 after 5, 7 and 9 days in culture compared to NC monolayers (Fig. 3c). We hypothesized that the difference observed in TEER measurements and FITC-PEG 400 could reflect either barrier function and/or maturation impairment, both suggested by the gene expression data set observed in organoids (Fig. 1c–e). Consistent with the gene expression data the immunofluorescence staining of the ZO1 showed less deposition of this protein along the cell perimeter of CD monolayers (Fig. 3d), suggesting defects in barrier deposition.

We further investigated the ability of the cell monolayers to differentiate in absorptive and secretory cells (see Methods). We evaluated the expression of lysozyme (LYZ), sucrase isomaltase (SI), mucin-2 (MUC2) and chromogranin A (CHGA) genes expressed respectively by Paneth’s cells, enterocytes, goblet’s cells and neuroendocrine cells. We found that LYZ was not differentially regulated in the two samples sets, neither was influenced by culturing conditions (Fig. 3e). As expected, the differentiating media promoted the maturation of enterocytes, goblet cells, and enteroendocrine in both NC and CD (Fig. 3f–h). Nevertheless, we observed a significant upregulation of MUC2 and CHGA expression at baseline and upon differentiation in CD monolayers when compared to NC cell monolayers (Fig. 3g,h).

### Gliadin increased intestinal permeability and triggered the release of pro-inflammatory cytokines in CD monolayers only

Gliadins have been shown to have immunomodulatory and gut-permeating functions^4^. A peptic-tryptic digest of α-gliadin (PTG) induced a significant increase in intestinal permeability in both biopsies and adenocarcinomas cell lines^5,6^, and in pro-inflammatory cytokine secretion^8,42^. We sought to study the response of CD and NC monolayers to PTG. The PTG was generated as previously reported^43^ and baseline cytotoxicity (Supplementary Fig. S3c) and endotoxin content was evaluated (see Methods).

**Figure 4:**
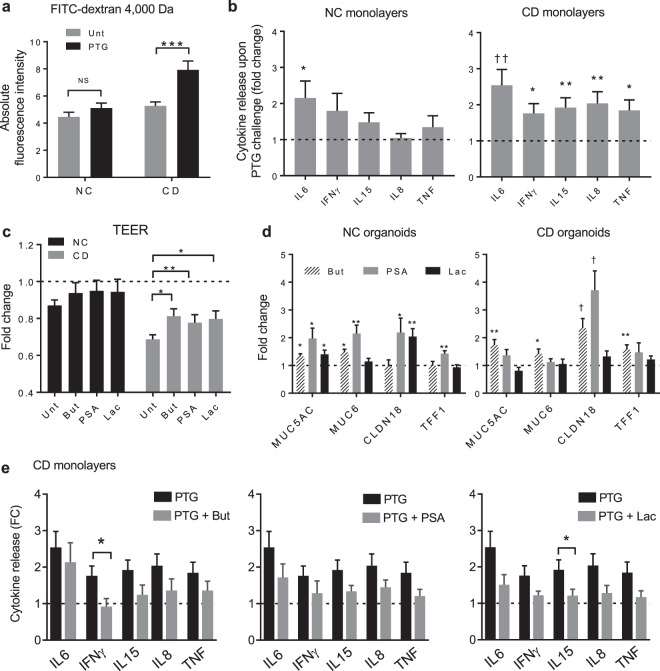
Effects of gliadin, butyrate, PSA and lactate on barrier function and pro-inflammatory cytokines. (**a**) Absolute fluorescent intensity of FITC-dextran 4,000 Da recovered in the basolateral side of confluent monolayers after 4 h incubation with 1 mg/mL pepsin-trypsin digested-gliadin (PTG) or media control (Unt). Data represent average ± SEM. ***p < 0.001; Mann-Whitney test; 21 to 30 replicates for n = 5 non-celiac (NC) and n = 6 celiac (CD) patients. NS: not-significant. (**b**) Secreted cytokines measured by ultra-sensitive multiplex electrochemiluminescence in the basolateral supernatants of non-celiac (NC) and celiac (CD) monolayers upon 4 h challenge with 1 mg/mL PTG. Fold change calculated relative to untreated monolayers. Data represent average ± SEM. *p < 0.05, **p < 0.01, two-side unpaired t-test. ^††^p < 0.01, Mann-Whitney test; 10 to 15 replicates for n = 5 NC and n = 6 CD patients. (**c**) Fold change of transepithelial electrical resistance (TEER) measured in non-celiac (NC) and celiac (CD) confluent monolayers after 48 h treatment with butyrate (But), polysaccharide A (PSA) or lactate (Lac), expressed as fold change of TEER reading at time 0. Unt: untreated. Data represent average ± SEM. *p < 0.05, **p < 0.01, Mann-Whitney test; 44 to 102 replicates for n = 5 NC and n = 6 CD patients. (**d**) Expression of genes related to gut barrier function assessed by quantitative RT-PCR in epithelial organoids derived from non-celiac (NC) and celiac (CD) patients, after 48 h treatment with butyrate (But), polysaccharide A (PSA) or lactate (Lac). Data represent average expression relative to untreated ± SEM. *p < 0.05, **p < 0.01, two-side unpaired t-test; ^†^p < 0.05, Mann-Whitney test. 8 to 12 replicates for n = 4 NC and n = 4 CD patients. (**e**) Secreted cytokines measured by ultra-sensitive multiplex electrochemiluminescence in the basolateral supernatants of celiac (CD) monolayers. Monolayers were 48 h pre-treated with microbiota derived-bioproducts butyrate (But), polysaccharide A (PSA) or lactate (Lac) and subsequently challenged with 1 mg/mL PT-gliadin (PTG) for 4 h. Fold change (FC) calculated relative to untreated monolayers. Data represent average ± SEM. *p < 0.05, Mann-Whitney test (PTG vs. PTG pretreated with the bioproducts); 12 to 15 replicates for n = 6 (CD) patients.

We aimed at evaluating the effect of PTG on tight junction functionality by challenging the monolayers derived from NC and CD organoids. PTG was apically administered to the monolayers for 4 hours. We found that the treatment with PTG increased the paracellular permeability of CD monolayers, based on FITC-dextran 4,000 Da paracellular passage (Fig. 4a), whereas did not affect paracellular permeability of NC monolayers. Furthermore, we evaluated the immune response of PTG treated monolayers. We selected a panel of five pro-inflammatory cytokines to be assessed in the basolateral supernatant. Based on previous reports we evaluated IL8 and IL6, which were found to be significantly upregulated in biopsies or Caco-2 cells treated with PTG^8,42^. We expanded our analysis to other pro-inflammatory cytokines critical for CD pathogenesis including IL15, which is relevant for the recruitment of intraepithelial lymphocytes^9^ and TNF and IFNγ, which are detrimental to intestinal integrity^44^. We found that PTG significantly stimulated the secretion of IL6 in both NC and CD monolayers, whereas it triggered a significant release of IFNγ, TNF, IL15 and IL8 cytokines only in the CD monolayers (Fig. 4b). The PTG negative control (PTG-CT) did not affect the release of any of the analyzed cytokines (see Methods; Supplementary Fig. S3b).

### Microbiota-derived bioproducts modified CD epithelium response

Intestinal dysbiosis has been reported in CD and other intestinal inflammatory diseases^13,45^. Additionally, a microbiota imbalance has been claimed to be associated with CD onset^15^.

We aimed at evaluating whether microbiota-derived molecules (bioproducts) could exert a protective role on the epithelial functions critical to the development of CD. Based on previous observations^15^ and as a proof of concept, we decided to analyze the effect of butyrate, lactate, and polysaccharide A (PSA). This last bioproduct, which holds recognized immunoprotective activity^46,47^, was derived from *Bacteroides fragilis*. The concentration of metabolites used reflected physiological levels in non-dysbiotic individuals relatively to butyrate and lactate^15^. PSA was evaluated at concentration previously adopted in other cellular models^48^.

CD and NC organoids were treated for 48 h with the three bioproducts, and their effect on epithelial barrier functionality was evaluated by measuring TEER and gene expression. Initially, we found that all three of the bacteria-derived bioproducts significantly ameliorated TEER changes in CD (Fig. 4c). Thus, we analyzed the expression of genes related to gut barrier function, found altered in CD data set (Fig. 1b,c). We found that butyrate significantly upregulated the expression of MUC5AC, MUC6, TFF1 and CLDN18, in CD organoids. PSA significantly increased the expression of CLDN18 only, while lactate did not alter the expression of the analyzed gut barrier function-associated genes. Similar regulation of genes was observed in NC organoids (Fig. 4d).

Finally, we analyzed the effect of the bacterial bioproducts on cytokines released by the CD monolayers challenged with gliadin. We observed that all bioproducts exerted a global protective effect by reducing the pro-inflammatory cytokine secretion triggered by PTG. However, only lactate and butyrate significantly reduced the secretion of IL15 and IFNγ cytokines, respectively in CD (Fig. 4e). In NC monolayers, IL6 secretion was also reduced by all the bioproducts however not significantly (Supplementary Fig. S3d).

## Discussion

With this study, we have provided novel functional and molecular data on the contribution of the intestinal epithelium to CD pathogenesis. We have paid particular attention to the effects triggered by the interaction between the host epithelium and environmental factors involved in CD pathogenesis, including causal relationship between microbial effectors (bacterial bioproducts), environmental triggers (gluten), and gene variants (host) leading to CD.

The intestinal epithelium plays a recognized and critical role in the development of CD and other autoimmune diseases^49^. It provides a physical barrier against pathogens and toxins, senses the presence of luminal pathogens, coordinates antigen trafficking, modulates the response of resident immune cells, and dynamically interacts with gut microbiota^50^.

To pursue our objectives, we have generated organoids from the epithelial component of the duodenum of NC and CD patients. By whole transcriptome analysis we have identified 472 genes differentially regulated between the samples sets. Furthermore, we have shown that selected genes identified by RNA-seq in the active CD organoid samples were similarly differentially regulated in whole CD biopsies. These data are consistent with previous observations^30,51^ and provide additional evidence that organoids recapitulate the gene expression profile of the epithelium of origin. Among the differentially expressed gene sets, we have identified novel genes associated with relevant epithelial functions potentially related to CD pathogenesis, namely gut barrier, regeneration (stem cells) and innate immunity. Relative to barrier function-associated genes, we have identified the tight junction protein CLDN18 and the mucin components (MUC6 and MUC5AC) that were significantly downregulated in CD epithelium. Moreover, we found that, consistent with previous studies, CD organoids have upregulated expression of pore-forming CLDN2^29^, downregulated expression of the tight junction protein ZO1^28^ and the mucus stabilizer factor TFF1^51^. These gene expression data suggest a constitutive defect of barrier functionality in CD, as previously hypothesized^29,52,53^. In line with the molecular data, we have also demonstrated that CD epithelia have a higher paracellular permeability at baseline, in the absence of a challenging agent (gluten).

Furthermore, we have shown that epithelia from active CD have impaired capabilities to proliferate, but not altered apoptosis at baseline. Our data suggest that on average CD organoids have less cells undergoing S phase, that reflects on slower growth, compared to control. We have also observed a modest increase in the percentage of cells in G2/M phase in CD. Whether the impaired proliferation is the consequence of a slower mitosis needs further investigation. Our data generated *in vitro* are consistent with longitudinal studies on mucosal healing in patients on a GFD. Although most celiac patients are able to fully recover the tissue damage after long term GFD (8 years), about 13% show only mucosal improvement and 6% had persistent villus atrophy^54^. What are the factors that promote the “resetting” of the stem cells back to normal, upon the acute insult, leading to mucosal recovery are not known yet and represent an exciting field for further investigation. Understanding these processes might be beneficial for those patients that do not fully recover from the mucosal damage even after long term GFD.

We have also reported that the analyzed cohort of active CD organoids has altered capabilities of differentiating. CD organoids overexpress stem-related and Paneth cells specific genes, suggesting that organoids from active CD patients express a crypt-like phenotype, in other words, contain more immature cells and that the crypt-like phenotype is independent on the insult (gluten).

The characterization of the cell monolayers has further corroborated the evidence of altered cell maturation in CD. Both NC and CD differentiated the absorptive lineage comparably. However, we identified significant differences in the secretory lineage signature. We observed upregulation of secretory-specific goblet’s cell marker MUC2. These data are consistent with previous observations on isolated intestinal epithelium from active CD^51,55^. Similarly, we found upregulation of CHGA in CD monolayers. CHGA is specifically expressed by enteroendocrine cells and to our knowledge its upregulation in CD has not being previously reported.

Finally, we have explored the immune profile of CD epithelia. We uncovered novel immune-related genes not previously identified, such as CCL25 and IL37 significantly upregulated in CD organoids. The chemokine CCL25 promotes the recruitment of immune cells such as macrophages^56^ and dendritic cells^57^. CCL25 overexpression has been reported in ulcerative colitis^58^. While its involvement in CD has been hypothesized^59^, to our knowledge, it has never been previously shown. We also uncovered that CD organoids overexpressed IL37, an anti-inflammatory cytokine related to chronic inflammation and autoimmune diseases^60^. Of note, IL37 has been found upregulated in systemic lupus erythematosus^61^, inflammatory bowel disease^62^, and rheumatoid arthritis^63^, and it has been shown to inhibit both innate and adaptive immunity^64^. We can speculate that the upregulation of IL37 might reflect a general, adaptive protective mechanism, common to multiple autoimmune diseases, to compensate for a system more prone to inflammation. Moreover, both IL37 and CCL25 upregulation were also confirmed in CD biopsies. Finally, we also confirmed the upregulation of NLRP6, initiator of the inflammasome complex formation^65^ that was previously found overexpressed in epithelial cells isolated from biopsies of CD active patients^51^.

Because we provided evidence that organoids faithfully recapitulated the epithelium of origin, we aimed at employing organoid-derived monolayers to gain insights on the functional responses of epithelia from CD patients to gliadin. Gliadins are prolamins responsible for cytotoxic, immunomodulatory, and gut-permeating activities of gluten^4^. Previous studies have shown that a peptic-tryptic digest of gliadin (PTG) augmented intestinal permeability in Caco-2 monolayers^5^ and human intestinal explants^6^. In this study, we have adopted a similar PTG challenge for monolayers from NC and CD organoids. Comparable HP2 genotypes ensured that there was not genetic bias in terms of the tight junctions’ modulation by zonulin across the sample sets tested^5,39^. Our results showed that PTG increased intestinal paracellular permeability in CD monolayers only. Conversely, PTG did not affect permeability in NC-derived monolayers. These data provide, for the first time, evidence of significant epithelial functional differences between CD and NC epithelia in response to gliadin, an observation not previously anticipated based on data generated in adenocarcinoma cell lines^5^.

We have further investigated the profile of cytokines secreted by the epithelia exposed to PTG. Multiple studies have shown that gliadin promotes the secretion of pro-inflammatory cytokines. Intestinal epithelial cells Caco-2/TC7 that were stimulated with PTG secreted IL6^42^, whereas, contrasting data have been generated relative to IL8^8^.

Consistent with previous observations^5^, we found that PTG triggered the release of IL6 in both CD and NC monolayers. However, PTG caused significant secretion of pro-inflammatory cytokines IL8 in CD epithelia only. We also explored the secretion of other biologically relevant cytokines like IL15, IFNγ and TNF. The cytokine IL15 has been shown to be overexpressed in CD patients^66^ and is expressed by intestinal epithelia^67^. Importantly, IL15 plays a central role in CD pathogenesis^68^ by promoting dysregulation of multiple immune mechanisms, by recruiting intraepithelial lymphocytes^9^ and activating Th1 cytokine production (IFNγ and TNF)^44^. We found that IFNγ, TNF, and IL15 were significantly more secreted in CD organoid-derived monolayers only upon gliadin stimulus. These last data further support functional differences between CD and NC epithelia relative to PTG challenge. Importantly, our results provide novel evidence for the use of CD patient-derived organoids as a tool for the identification and the study of agents targeting CD-specific reactions to gluten.

Finally, we sought to establish whether other environmental factors, along with gluten and host genetics, could contribute to or sustain the pathogenesis of the disease. A large number of studies have suggested the involvement of gastrointestinal microbiota in the pathogenesis of CD^45^ and other autoimmune diseases^69^. Nevertheless, most of these studies merely showed correlation and not mechanistic causation between microbiota composition and disease status. In this study, we selected three bacterial bioproducts to be analyzed: butyrate, lactate, and PSA from *B*. f*ragilis*. Our rationale was based on previous observations showing significant alterations of the bacteria phyla producing them and/or their relative abundance (butyrate and lactate) in the preclinical phase of CD^15^.

The effects of butyrate on eukaryotic cells have been extensively reported. Specifically, butyrate plays an important regulatory role on transepithelial ion transport^70^, ameliorates mucosal inflammation^71^, reduces IFNγ signaling^72^, and ameliorates loss of gut barrier function by increasing the sealing tight junction proteins^73^. Lactate is the major product of several bacterial families with probiotic properties including *Lactobacilli* and *Bifidobacteria*^74^. These bacteria have been shown to be beneficial for the prevention and treatment of gastrointestinal diseases including CD^75^. Specifically, Lactobacillus protects epithelial cells against the harmful effects of TNF and IFNγ^76^. Finally, studies on PSA suggest that it prevents intestinal inflammatory disease by promoting the differentiation of regulatory cells (Treg) and enhancing IL10 production^77^. In necrotizing enterocolitis (NEC), PSA inhibits IL1β-induced IL8 inflammation in a fetal intestinal epithelial cell line (H4 cells)^47^.

Given the important role that these three bacterial bioproducts play in maintaining intestinal homeostasis, we evaluated their effects on CD epithelial barrier and immune functions at the baseline and upon gluten challenge. We found that treatments with all the bacterial bioproducts improved the barrier functionality of CD epithelia. These functional data correlated with the upregulation of mucins (MUC5AC and MUC6), TFF1 and CLDN18 expression, genes that were found significantly downregulated in the CD organoid cohort. Consistent with previous data, butyrate was the most biologically active in promoting barrier function. Relative to the CD innate immune response to gluten, we found that lactate and butyrate significantly reduced the gliadin-induced secretion of IL15 and IFNγ. Taken together, our observations indicate that the investigated bacterial bioproducts have a positive impact on barrier function and might exert a protective role in terms of mitigating an immune response to gliadin.

To summarize, our study has provided evidence that CD epithelium express stable transcriptional differences potentially relevant to develop and/or sustain the enteropathy. The molecular data have been further supported by our functional observations in CD-derived monolayers. These novel findings proved further insight in the early steps of CD pathogenesis that were poorly defined so far amid the lack of robust experimental models. Furthermore, we have shown that microbiota-derived bioproducts are promising factors to improve the barrier functionality of the epithelium and to reduce the gliadin-induced pro-inflammatory profile in CD. Finally, we have validated the use of *in vitro* patient-derived organoids to model CD pathogenesis to further study CD treatment and prevention.

## Methods

### Isolation of duodenal crypts and establishment of culture of human organoids

Duodenal biopsies from NC (n = 5) and CD (n = 6) patients were collected by upper endoscopic procedure performed for routine diagnosis or clinical follow-up. CD diagnosis was confirmed by histopathologic analysis and levels of tTG IgA blood antibodies, whereas NC patients presented normal duodenal mucosa with no evidence for CD. Patients in both groups had comparable average age (NC = 56.2 ± 13.3 years and CD = 54.6 ± 20.6 years) and gender distribution, with female donors in the majority (70%).

After collection, biopsies were immediately placed in ice-cold DMEM/F12 complete medium and processed for isolation of intestinal epithelial cells or frozen for further gene expression analysis. The isolation of intestinal epithelial cells was performed as previously described^18,19,41^. The duodenal organoids’ culture was passaged every 7–9 days using a standard, trypsin-based cell dissociation protocol. About 2*10^6^ cells/mL singles were re-plated in matrigel supported by L-WRN/ISC medium^19^ and kept at passages ranging from P5 to P22.

### Preparation of reagents

A peptic-tryptic digest of gliadin (PTG) was generated by sequential digestion with pepsin and trypsin as previously described^43^ with the following modification. Briefly, gliadin from wheat (#G3375, Sigma-Aldrich) was dissolved in 5% formic acid at 1 mg/mL and digested with pepsin (#P6887, Sigma-Aldrich) at an enzyme/substrate ratio of 1/100, with shaking for 2 hours at 37 °C. The gliadin peptic digest was lyophilized and further dissolved in 100 mM ammonium bicarbonate at 1 mg/ml. Trypsin (#T9201, Sigma-Aldrich) was added at an enzyme/substrate ratio of 1/100 and incubated with shaking for 4 hours at 37 °C. The reaction was stopped by boiling for 5 minutes. Identical conditions (minus substrate gliadin) were applied to generate a PTG negative control (PTG-CT) that was employed in some experiments to test the biological activity of the PTG-CT (Supplementary Fig. S3a,b). The PTG and PTG-CT were centrifuged at 4,000 rpm for 10 minutes, aliquoted, lyophilized, and stored frozen at −80 °C. PTG was resuspended at 10 mg/mL and used at a final concentration of 1 mg/mL, as previously reported^5,39^.

Sodium L-lactate (#L7022, Sigma-Aldrich), and sodium butyrate (#B5887, Sigma-Aldrich) were resuspended at 1.5 mg/ml and 1.0 mg/ml respectively, in DMEM/F12, aliquoted and stored at −80 °C, and were used at a working concentration of 1.5 μg/mL and 1.0 μg/mL, respectively. Polysaccharide A (PSA) was purified from *Bacteroides fragilis* NCTC 9343 and provided by Dr. Dennis Kasper, Department of Microbiology and Immunobiology, Harvard Medical School, Boston, MA, USA, prepared as previously described^78^. PSA was resuspended in sterile PBS at 100 mg/ml, stored at −80 °C, and used at a working concentration of 100 μg/mL.

PTG, PTG-CT, lactate, butyrate, and PSA were tested at working concentrations for endotoxin levels using the Limulus Amebocyte Lysate assay (QCL-1000, Lonza, USA) according to manufacturer’s procedures. All the reagents had an endotoxin level ≤1.5 EU/mL. Lactate dehydrogenase (LDH) assay was employed to test the cytotoxicity of the generated reagents (CytoTox 96 Non-Radioactive Cytotoxicity Assay, #G1780, Promega, USA). Similar levels of LDH were detected for untreated and treated monolayers with the bacterial derived-molecules or PTG or PTG-CT (Supplementary Fig. S3c).

### Duodenal organoid-derived monolayers and experimental procedures

Organoid-derived monolayers were established by seeding 1*10^5^ single cells in 100 μl L-WRN/ISC medium per well on an uncoated polyester membrane transwell inserts with a 0.4 μm pore size (24 well plate, #3470, Corning, USA). The L-WRN/ISC medium was freshly supplemented with Y-27623 Rock inhibitor and changed every other day. TEER measurements and microscope direct observation were employed to monitor confluence. In order to promote cell differentiation and maturation, the confluent monolayers were apically treated with 5 μM DAPT (#565784, Calbiochem) in DMEM/F12 for 48 hours^19^, whereas basolateral media contained L-WRN/ISC medium only. In some experiments, monolayers were apically pre-treated, along with DAPT, with the following: lactate, butyrate, or PSA for 48 hours, followed by media change DMEM/F12 and subsequently challenged with PTG at 1 mg/mL^5^ for 4 hours. Basolateral supernatant was collected for analysis. Experiments were performed at least three times in triplicates.

### Measurement of integrity and paracellular permeability of the monolayers

TEER was evaluated during development of the monolayers and after PTG challenge as a quantitative measure of barrier integrity^79^. A dual planar electrode instrument (Endhom Evom, World Precision Instruments, USA) was employed according to manufacturer’s directions. Data were expressed as resistance multiplied by the area (Ω*cm^2^).

Paracellular permeability was evaluated by measuring the diffusion of two different molecular size neutral molecules: FITC-dextran, molecular weight of 4,000 Da (#FD4 Sigma-Aldrich) and FITC-PEG of 400 Da (#PG1-FC-400, Nanocs, USA). FITC-dextran or FITC-PEG was added apically at 1 mg/ml and measured in the basolateral media after 4 hours by spectrophoto fluorimetry (Synergy 2, Biotek, USA) (485/528 nm excitation/emission wavelength), as previously reported^80^.

### Organoids’ growth evaluation

To examine the organoids growth over time, NC and CD organoids were plated at 5*10^4^ cells/mL and cultured for 7 days as previously described. Multiple fields per sample were acquired at 2, 4 and 7 days after plating using a bright field direct microscope (Invertoskop 40 C, Zeiss, Oberkochen, Germany). We calculated the area of the organoids (μm^2^) in the captured microfield using ImageJ Software (National Institute of Healthy, USA) and then averaged the areas based on the total number of the measured organoids.

### Immunofluorescence staining

Cells were fixed in 4% paraformaldehyde and directly stained based on standard protocols^19^. The monolayers and organoids images were acquired using the microscope Nikon C2 confocal and Nikon Eclipse 80i (Nikon, USA), respectively. The cells were co-stained with 4′-6′-diamino-2phenylindole 1:1000 (DAPI) nuclear marker (blue). Antibodies: ZO1 Alexa Fluor 488 conjugate 1:100 (#339188, Invitrogen, USA) and cleaved caspase-3 (Asp175) 1:200 (#9661, Cell Signaling Technology, USA).

### Propidium iodide staining

NC and CD organoids were collected, trypsinized and fixed in ethanol 66% accordingly to standards protocols. Propidium iodide staining was performed with Propidium Iodide Flow Cytometry Kit for Cell Cycle Analysis (#139418, Abcam, USA) following the manufacturer instructions. 2*10^4^ cells per samples were acquired and analysis was performed on gated cells that excluded debris, apoptotic cells and doublet cells (Supplementary Fig. S2c,d).

### Fluorescence activated cell sorting and analysis (FACS)

Staining for Ki67 and phospho-histone H3 (H3P) was performed on trypsinized cells from NC and CD organoids previously fixed in formaldehyde 4% and permeabilized in methanol 90%. Cells were then washed in PBS, incubated with anti-Ki67 antibody (FITC-conjugate, #16667, Abcam) and H3P (Pacific Blue conjugate, #8552, Cell Signaling Technology, USA) in Bovine Serum Albumin 0.5% in PBS (#A7906, Sigma-Aldrich) and washed twice. 10^5^ cells per samples were acquired and analysis was performed on gated cells that excluded debris and double positive (Ki67^+^H3P^+^) representative of cells that entered mitosis. Data were acquired with BD FACS Calibur flow cytometer instrument. Analysis was performed with BD CellQuestPro software.

### Gene expression analysis

RNA extraction from organoids, monolayers and duodenal were carried out in TRIzol reagent and total RNA was purified using Zymo-Spin column (#R2050, Zymo Research, USA), according to manufacturer’s protocol. cDNA was generated from RNA using the Maxima H minus first strand cDNA synthesis kit (#K1652, Thermo Fischer Scientific). SYBR Green reagent (Applied Biosystems/Life Technologies, US) and CFX Connect Real-time PCR detection system (Bio-Rad, USA) were used for gene expression analysis (quantitative RT-PCR). The 18S gene expression was used as an internal control. The results were expressed as the fold change over sample control applying the C_T_ method (${2}^{-{\rm{\Delta }}{\rm{\Delta }}{C}_{T}})$^81^. The oligonucleotide primers used were designed by the MGH primer bank (Boston, MA, USA) and synthesized by IDT (San Jose, CA, USA) or purchased from Qiagen, (Limburg, Netherlands) (Table 1).

**Table 1:** Oligonucleotide primers for quantitative RT-PCR analysis.

Gene	Forward	Reverse
18S	AGAAACGGCTACCACATCCA	CCCTCCAATGGATCCTCGTT
ASCL2	GCGTGAAGCTGGTGAACTTG	GGATGTACTCCACGGCTGAG
CCL24	ACATCATCCCTACGGGCTCT	CTTGGGGTCGCCACAGAAC
CCL25	GGCCCTCATGCTGTAAAGAAG	TGCTGATGGGATTGCTAAACTT
CHGA	TAAAGGGGATACCGAGGTGATG	TCGGAGTGTCTCAAAACATTCC
CLDN18	ACATGCTGGTGACTAACTTCTG	AAATGTGTACCTGGTCTGAACAG
CLDN2	PPH02779A (Qiagen)	
DEFA5	AGACAACCAGGACCTTGCTAT	GGAGAGGGACTCACGGGTAG
Haptoglobin (HP)	TTTCTGGCTGCTAAGTGG	AATGTCTTTCGCTGTTGC
IL37	TTCTTTGCATTAGCCTCATCCTT	CGTGCTGATTCCTTTTGGGC
LGR5	PPH13346A (Qiagen)	
LYZ	CTTGTCCTCCTTTCTGTTACGG	CCCCTGTAGCCATCCATTCC
MUC2	GCCAGCTCATCAAGGACAG	GCAGGCATCGTAGTAGTGCTG
MUC5AC	CAGCACAACCCCTGTTTCAAA	GCGCACAGAGGATGACAGT
MUC6	CTGCCCTATACCAGCAATGGA	CTGACCCATGTACTTCCGCTC
MYC	GTCAAGAGGCGAACACACAAC	TTGGACGGACAGGATGTATGC
NLRP6	CCTACCAGTTCATCGACCAGA	CTCAGCAGTCCGAAGAGGAA
OLFM4	ACTGTCCGAATTGACATCATGG	TTCTGAGCTTCCACCAAAACTC
PCNA	CCTGCTGGGATATTAGCTCCA	CAGCGGTAGGTGTCGAAGC
SI	TCCAGCTACTACTCGTGTGAC	CCCTCTGTTGGGAATTGTTCTG
SMOC2	ATGACGACGGCACCTACAG	TCGCGTTGGGGTAACTTTTCA
TFF1	CCCTCCCAGTGTGCAAATAAG	GAACGGTGTCGTCGAAACAG
TNFRSF25	PPH00349B (Qiagen)	
TP53	ACTTGTCGCTCTTGAAGCTAC	GATGCGGAGAATCTTTGGAACA
ZO1	CAACATACAGTGACGCTTCACA	CACTATTGACGTTTCCCCACTC

RNA-seq analysis: total RNA isolated as described was subjected to polyA selection, followed by NGS library construction using NEBNext Ultra Directional RNA Library Prep Kit for Illumina (New England Biolabs, USA). Sequencing was performed on an Illumina HiSeq 2500 instrument and reads were mapped to the human reference genome (hg19 build) using STAR^82^, resulting in a range of 30–50 million aligned single-end 50 bp reads per sample. Read counts over transcripts were calculated using HTSeq v.0.6.0^83^ based on the most current Ensembl annotation file for hg19. Genes with RPKM (Reads Per Kilobase per Million mapped reads) greater than 1.0 were considered transcriptionally active. Functional annotation analysis was performed on differentially expressed genes (two-fold change and FDR < 0.05 value) using DAVID v6.7 [https://david-d.ncifcrf.gov/]^22,23^. Functional gene set enrichment analysis (GSEA) was performed on the whole-transcriptome expression values using GSEA^24^ comparison against Hallmark gene sets with default parameters and a nominal p-value cutoff of 0.01.

### Haptoglobin (HP) genotyping

DNA isolation was performed by silica-based spin-column (#69504, Qiagen, USA), according to manufacturer’s instructions. Haptoglobin 2 genotyping was performed using specific primers (Table 1) designed in exon 2 and exon 5 of HP1 corresponding to exons 2 and 7 of HP2^84^ amplified by high fidelity PCR system (Arktik Thermal Cycler, Thermo Fisher Scientific). After PCR, the amplicons were run on a 1% agarose gel and read under a UV bulb (GeneFlash, Syngene, USA). The band size difference allowed differentiation of the two genotypes (HP1: 2.5 kb and HP2: 5.3 kb).

### Cytokine analysis

A pro-inflammatory panel of cytokines including IL6, IL8, IL15, TNF, and IFNγ was evaluated in the basolateral medium of treated monolayers using a multiplex electrochemiluminescence assay (#K15067L-2, MesoScale Discovery, USA), according to manufacturer’s instructions. The data analysis was performed using the MSD Discovery Workbench 4.0 software.

### Statistics

Analysis of RNA-seq data was performed using EdgeR package (version 3.8.6)^85^ based on the criteria of more than a two-fold change in expression value and FDR (Benjamini-Hochberg) less than a 0.05 value and the hypergeometric enrichment test. All other statistical analyses were performed using the software GraphPad Prism 7.0 (GraphPad Software). Normal distribution of the data set was evaluated using the D’Agostino & Pearson test. Parametric data were analyzed by Student’s t-test and nonparametric variables by Mann–Whitney test (2 groups). Data represent average ± SEM. A significance level of 0.1% (p < 0.001), 1% (p < 0.01) and 5% (p < 0.05) was adopted.

### Study approval

All protocols and samples collections were approved by the Massachusetts General Hospital Institutional Review Board (#2014P000198) and performed in accordance with relevant guidelines and regulations. All patients gave written informed consent prior to study inclusion.

## Supplementary information

Supplementary Information

Supplementary Table S1

Supplementary Table S2

## Data Availability

RNA-seq data generated for this study were uploaded at NCBI-GEO databank, Accession Number: GSE113492. All datasets generated and/or analyzed during the current study are available from the corresponding author on reasonable request.
