# Supplementary material for: Human gut derived-organoids provide model to study gluten response and effects of microbiota-derived molecules in celiac disease
Source: Sci Rep. 2019 May 7;9:7029. doi: 10.1038/s41598-019-43426-w (PMC6505524; doi:10.1038/s41598-019-43426-w)
Supplement: Supplementary file 1 — Supplementary Information [file 41598_2019_43426_MOESM1_ESM.pdf]

Supplementary Information

Human gut derived-organoids provide model to study gluten response and effects of microbiota-derived molecules in celiac disease

Rachel Freire[1,2], Laura Ingano[1], Gloria Serena[1,2], Murat Cetinbas[2,3], Anthony Anselmo[2,3,†], Anna Sapone[1,2,‡], Ruslan I. Sadreyev[2,3], Alessio Fasano[1,2,§], Stefania Senger*[1,2, §]

Supplementary Fig. S1

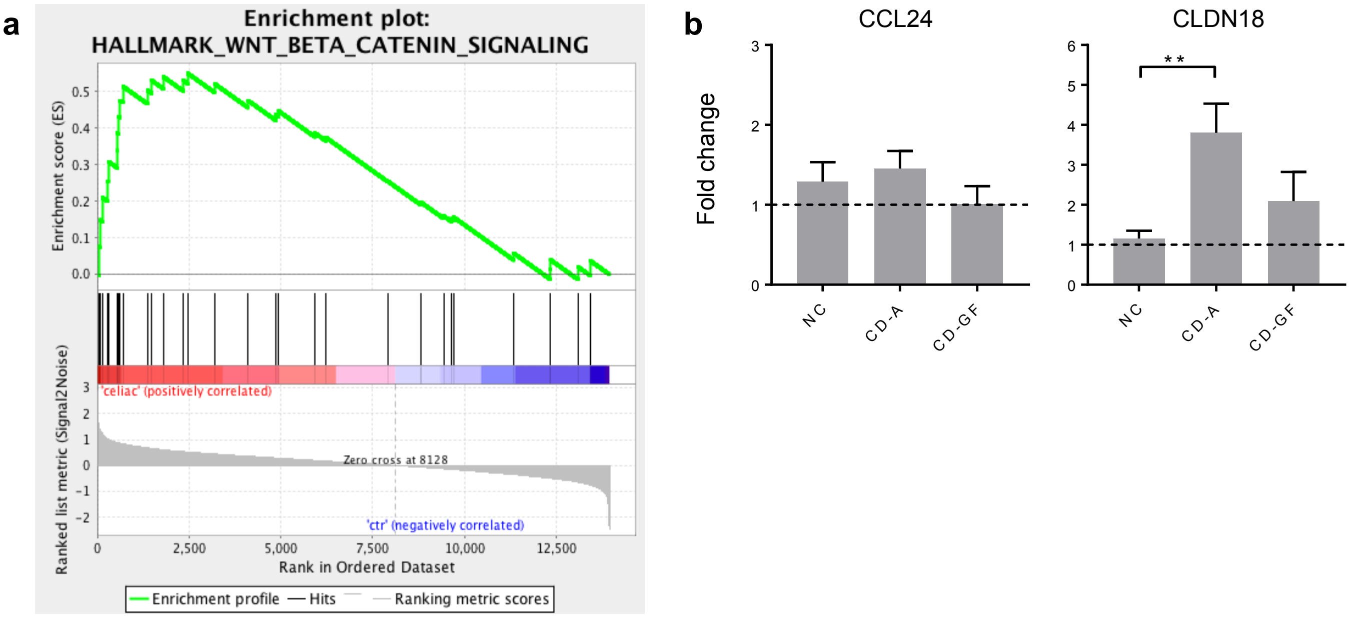

Supplementary Figure S1: GSEA enrichment analysis

a) Gene set enrichment analysis (GSEA) plots based on gene lists ranked by expression differences between active celiac (n=3) and non-celiac control (n=3) organoids. Wnt-Beta catenin signaling showed positive GSEA Enrichment Score (ES) (out of total 26 sets detected with nominal p-value < 0.01).

b) Gene expression assessed by qRT-PCR in human duodenal biopsies of non-celiac (NC, n=6-11), celiac patients with active disease (CD-A, n=17), and celiac patients in remission following a gluten-free diet (CD-GF, n=5-6) to validate the organoid model. Data represent average expression relative to NC ± SEM. **p<.01, Mann-Whitney test.

Supplementary Fig. S2

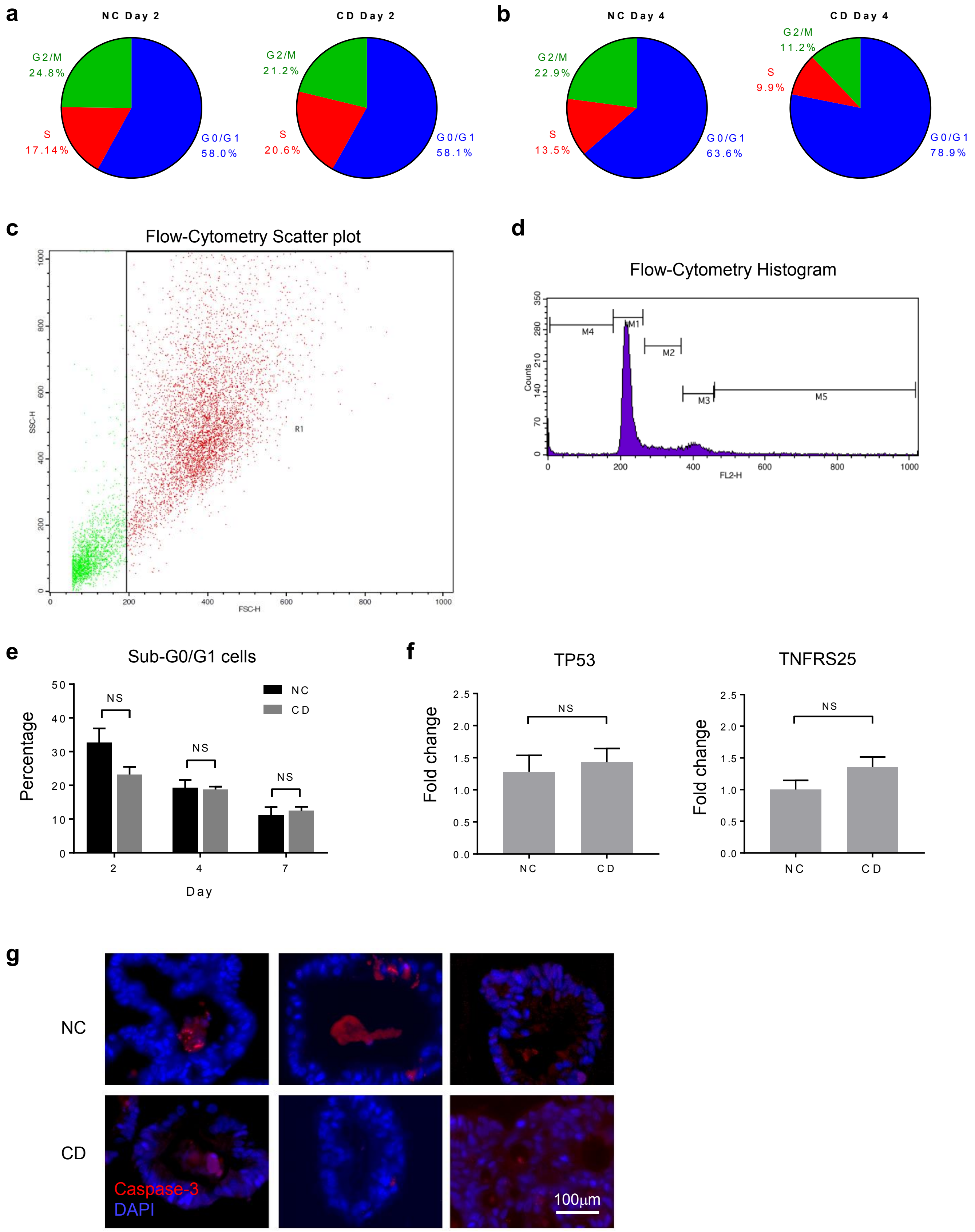

Supplementary Figure S2:

a-b) Pie charts representing the percentage of cells in G0/G1 phase (blue), S phase (red) and G2/M phase (green) in non-celiac (NC, n=4) and active celiac (CD, n=3) organoids as determined by propidium iodide staining after 2 days (a) and 4 days (b) in culture. Cells percentages were calculated out of gated cells that excluded debris, doublets and apoptotic cells.

c) Flow cytometry scatter plot representing gating strategy for PI staining cell-cycle analysis. R1 gated cells (red events) excluded cell-debris (green events) and were further analyzed based on PI intensity.

d) Exemplary flow cytometry histogram gating to identify respectively sub-G0/G1 (M4), G1/G0 (M1), S-phase (M2), G2/M (M3) by propidium iodide (PI) staining. The histogram included the cells gated in R1 (c).

e) Percentage of sub-G0/G1 phase cells in non-celiac (n=4) and active celiac (n=4) organoids as determined by propidium iodide staining. Data represent average ± SEM. NS: not-significant, two-side unpaired t-test.

f) Gene expression assessed by qRT-PCR in non-celiac (NC) and active celiac (CD) organoids. Data represent average expression normalized to NC control ± SEM. NS: not-significant, two-sided unpaired t-test. 12 to 18 replicates for n=5 NC and n=6 CD patients.

g) Immunofluorescence staining of activated Caspase-3 performed on n=3 non-celiac (NC) and n=3 active celiac (CD) organoids culture for 7 days. Cells were counter-stained with DAPI nuclear marker (blue). Scale bar: 100 μm.

Supplementary Fig. S3

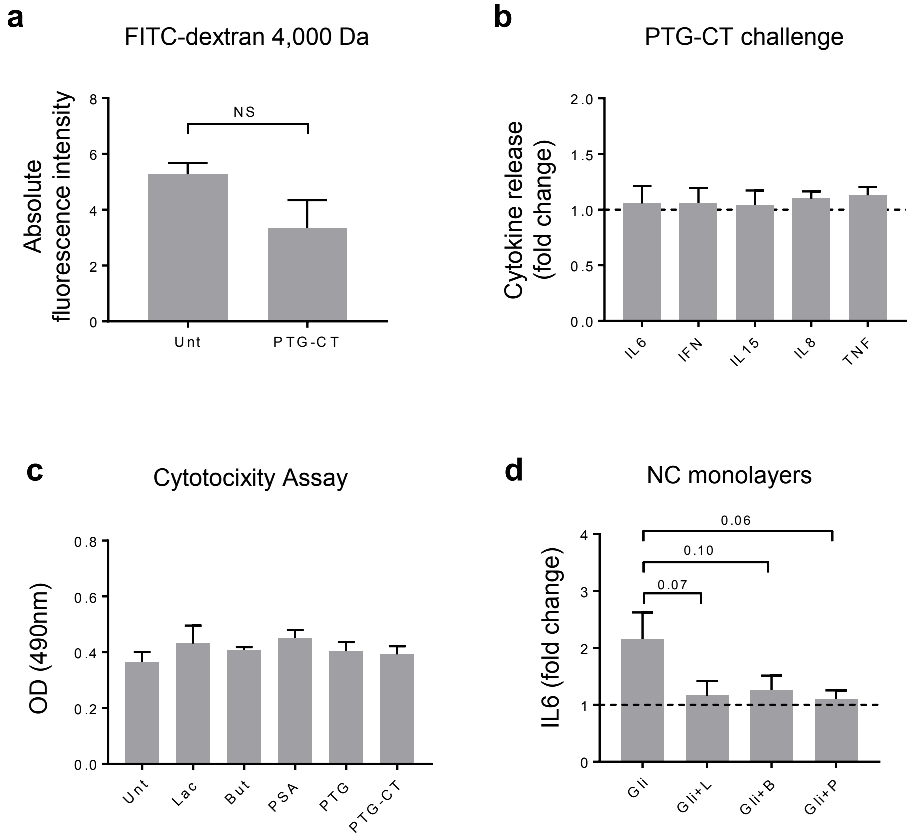

Supplementary Figure S3: Reagents cytotoxicity and biological activity of PT-gliadin negative control

a) Absolute fluorescence intensity of FITC-dextran (4,000 Da) recovered in the basolateral side of celiac (CD) confluent monolayers after 4h of incubation. Data represent average ± SEM. NS: not significant, Mann-Whitney test; 30 untreated and 14 PT-gliadin negative control (PTG-CT) observations for n=4 CD patients.

b) Secreted cytokines measured by ultra-sensitive multiplex electrochemiluminescence in the basolateral supernatants of celiac monolayers apically challenged with 1mg/mL PT-gliadin negative control (PTG-CT). Data represent average ± SEM. No statistical difference was observed. Mann-Whitney test; 4 observations representative for one celiac monolayer.

c) Lactate dehydrogenase-(LDH) based assay to evaluate cytotoxicity in organoid-derived monolayers exposed to the following reagents: media control (Unt: untreated); butyrate (But); lactate (Lac); PSA (Polysaccharide A); PTG (peptic-tryptic digested-gliadin); PTG-CT (PTG negative control).
